# Control of mitochondrial biogenesis and function by the ubiquitin–proteasome system

**DOI:** 10.1098/rsob.170007

**Published:** 2017-04-26

**Authors:** Piotr Bragoszewski, Michal Turek, Agnieszka Chacinska

**Affiliations:** 1 Laboratory of Mitochondrial Biogenesis, International Institute of Molecular and Cell Biology, Ks. Trojdena 4, 02-109 Warsaw, Poland; 2 Centre of New Technologies, Warsaw University, Banacha 2c, 02-097 Warsaw, Poland

**Keywords:** mitochondria, proteasome, ubiquitin, ubiquitin–proteasome system, proteostasis, protein biogenesis

## Abstract

Mitochondria are pivotal organelles in eukaryotic cells. The complex proteome of mitochondria comprises proteins that are encoded by nuclear and mitochondrial genomes. The biogenesis of mitochondrial proteins requires their transport in an unfolded state with a high risk of misfolding. The mislocalization of mitochondrial proteins is deleterious to the cell. The electron transport chain in mitochondria is a source of reactive oxygen species that damage proteins. Mitochondrial dysfunction is linked to many pathological conditions and, together with the loss of cellular protein homeostasis (proteostasis), are hallmarks of ageing and ageing-related degeneration diseases. The pathogenesis of neurodegenerative disorders, such as Alzheimer's disease and Parkinson's disease, has been associated with mitochondrial and proteostasis failure. Thus, mitochondrial proteins require sophisticated surveillance mechanisms. Although mitochondria form a proteasome-exclusive compartment, multiple lines of evidence indicate a crucial role for the cytosolic ubiquitin–proteasome system (UPS) in the quality control of mitochondrial proteins. The proteasome affects mitochondrial proteins at stages of their biogenesis and maturity. The effects of the UPS go beyond the removal of damaged proteins and include the adjustment of mitochondrial proteome composition, the regulation of organelle dynamics and the protection of cellular homeostasis against mitochondrial failure. In turn, mitochondrial activity and mitochondrial dysfunction adjust the activity of the UPS, with implications at the cellular level.

## 1. Introduction

### 1.1. Mitochondria

Mitochondria are multifunctional organelles in eukaryotic cells. Although mostly recognized as powerhouses because of their respiratory energy conversion, mitochondria perform various other essential functions. Mitochondria provide iron–sulfur cluster assembly, integrate anabolic and catabolic processes (including amino acid and lipid metabolism) and participate in cellular ion homeostasis and signalling pathways [1–6]. Their involvement in cellular metabolism makes mitochondria crucial even for eukaryotes that inhabit anaerobic environments, with only one recent example of the evolutionary loss of this organelle [7–9]. The perturbation of mitochondrial function results in cellular stress and often has devastating effects, including mitochondrion-related diseases in humans [2,10].

Mitochondria possess well-defined boundaries that are provided by two membranes that outline the organelle [11–14]. These membranes, external (outer mitochondrial membrane; OM) and internal (inner mitochondrial membrane; IM), surround two distinct aqueous subcompartments: the intermembrane space (IMS) and the mitochondrial matrix. The IM is further divided into an inner boundary membrane that is adjacent to the OM and is separated by crista junctions from invaginations that protrude into the matrix, called cristae. Mitochondria are organized into a dynamic network that is shaped by frequent fusion and fission processes [5,12,14].

**Figure 1.**
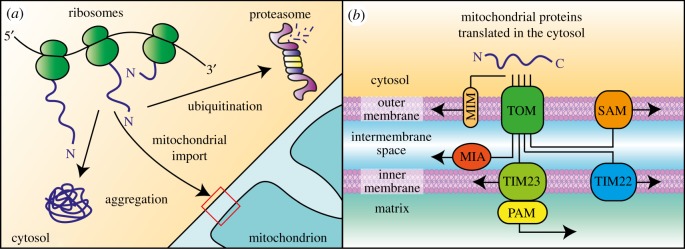
Cellular fate of mitochondrial precursor proteins translated in the cytosol. (*a*) The majority of mitochondrial proteins are encoded by genomic DNA, and their translation is executed outside mitochondria. After synthesis on ribosomes, mitochondrial proteins are transported to their destination inside mitochondria. In the case of failure of any of the processes that are involved in protein synthesis or transportation to the organelle, proteins are ubiquitinated and degraded by the proteasome or can form aggregates in the cytosol. (*b*) Schematic representation of mitochondrial protein translocation and sorting pathways. Precursor proteins that are synthesized in the cytosol cross the outer mitochondrial membrane by a common entry gate: the translocase of the outer membrane (TOM) complex. They are then routed by sorting pathways to their target location within mitochondria. Proteins that are destined to the outer membrane are built into the membrane by sorting and assembly machinery (SAM) or use the insertase of the mitochondrial outer membrane (MIM). Many proteins of the intermembrane space follow the mitochondrial import and assembly (MIA) pathway. Insertion into the inner mitochondrial membrane is mediated by translocases of the inner membrane TIM22 and TIM23. Matrix proteins use the TIM23 translocase coupled with the presequence translocase-associated motor (PAM).

To perform their functions, mitochondria need a set of proteins to build the mitochondrial proteome. The best-characterized proteomes of yeast and human mitochondria comprise approximately 1000–1500 different proteins, but the annotation of mitochondrial proteins is an ongoing process [15,16]. Mitochondria have their own genome and transcription and translation machinery [17,18]. However, a very limited number of mitochondrial proteins and peptides are synthesized inside the organelle. In humans, these consist of 13 protein subunits of respiratory complexes and small peptides with signalling functions. The vast majority of mitochondrial proteins are encoded by the nuclear genome and synthesized by cytosolic ribosomes in precursor forms. Subsequently, precursor proteins require active transport to their target location (figure 1*a*).

The complex architecture of the organelle and need to coordinate the assembly of multi-protein complexes with elements that are encoded in separate genomes makes mitochondrial biogenesis a challenging process. To ensure precise protein targeting, a versatile system integrates many protein sorting, translocation and folding machineries (figure 1*b*) [7,19–24]. The translocase of the outer membrane (TOM) is a multi-subunit complex that serves as the receptor and entry gate for the vast majority of mitochondrial proteins. Importantly, mitochondrial protein precursors need to be largely unfolded to pass translocases. After passing the OM via TOM protein-conducting channels, the proteins are routed to their final destination, which is encoded in their amino acid sequence. Precursors of β-barrel OM proteins use the sorting and assembly machinery (SAM) complex coupled with the TOM for their membrane insertion. Other OM proteins can use TOM coupled with the mitochondrial insertase of the outer membrane (MIM). For some OM-anchored proteins, translocation through the TOM is unnecessary. Most proteins of the IMS use the mitochondrial import and assembly (MIA) pathway, which combines protein import with oxidative folding. Protein oxidation by the MIA pathway leads to the formation of structural disulfide bonds in substrate proteins that are necessary for their functions but also provide a trapping mechanism for retention in the IMS. Precursor proteins that are directed to the IM use the translocases of the inner membrane, TIM22 or TIM23, for their membrane insertion. TIM22 governs the insertion of multi-pass transmembrane proteins, such as mitochondrial metabolite carriers and TIM translocase components. The pathway for protein import into the matrix is governed by the TIM23 complex, which pairs with the presequence translocase-associated motor (PAM) acting on the matrix side of the IM. TIM23 is also used by some proteins that are anchored in the IM by transmembrane domains (TMDs). Precursor proteins that use TIM23 contain N-terminal targeting sequences that are rich in positively charged amino acids. These targeting sequences use the electrochemical potential across the IM to initiate their translocation to the matrix, which is accomplished with the help of the adenosine triphosphate (ATP) hydrolysing motor. Upon import into the matrix, the proteins undergo proteolytic removal of their targeting signal, an important step in their maturation.

Proteins are constantly at risk of misfolding, becoming damaged and aggregating. Mitochondria possess machineries both to refold misfolded proteins and to degrade damaged ones [25,26]. Moreover, specific mechanisms allow mitochondria to segregate damaged proteins in vesicles that are delivered to the lysosome or vacuole for degradation [27–29]. However, the diversity/plasticity of the mitochondrial proteome and complexity of its biogenesis require integration with two major cytosolic protein degradation machineries: autophagy and the ubiquitin–proteasome system (UPS). Increasing evidence demonstrates that shaping the mitochondrial proteome by both cytosolic quality control pathways is crucial for cell fitness. Mitophagy is a mitochondria-specific type of autophagy, degrading damaged organelles in bulk. The UPS delivers a high level of selectivity by degrading specific protein targets one at a time. The UPS is responsible for the turnover of most cytosolic short-lived proteins. Thus, the UPS is able to provide both quality control and a regulatory mechanism.

The aim of this review is to summarize data on the various levels at which the UPS affects the mitochondrial proteome and the impact of UPS-mediated protein degradation on mitochondrial biology. We also discuss mitochondrial feedback that affects proteasome function, and thus impacts protein homeostasis (proteostasis) at the cellular level.

### 1.2. Ubiquitin–proteasome system

The proteasome is a dynamic multi-protein complex that exists in several variants [30–32]. The central part of the proteasome is a 20S core particle. This barrel-shaped structure is formed by four stacked heteroheptameric rings. Two inner β-rings have proteolytic activity, and two outer α-rings form a gated pore at both ends of the barrel. The confinement of proteases inside the 20S core structure provides an elementary regulatory mechanism. The core particle requires the docking of additional components or specific stimuli to open its gates. Regulatory or activating protein complexes can bind at both ends of the core particle barrel to tune its activity [32].

The specific degradation of ubiquitinated proteins is governed by the 26S proteasome. To form the 26S proteasome, the 20S core particle binds with 19S regulatory particles. The 19S regulatory particles provide the ability to recognize client proteins that are tagged with ubiquitin, and thus maintain the specificity of the degradation process. The 19S regulatory particles also contain a ring of AAA ATPases that participate in substrate transport to 20S core particles [32].

Protein substrates are tagged for degradation by the covalent attachment of ubiquitin, a small and strictly evolutionarily conserved protein [33–37]. This requires the coordinated action of three types of enzymes. First, the ubiquitin-activating E1 enzyme activates ubiquitin and transfers it to the ubiquitin-conjugating E2 enzyme. Second, the E2 enzyme cooperates with the E3 ubiquitin ligase to transfer ubiquitin to the substrate protein, usually to lysine residues. E3 proteins form a very diverse group. The human genome encodes 600–1000 different E3s. Such a large group of E3 proteins is required because they provide specificity for substrate selection. The attachment of one ubiquitin is called monoubiquitination. In the case of small proteins, it can be a sufficient signal for proteasomal degradation [38]. However, polyubiquitin chains are frequently built on the substrate. In such chains, each subsequent ubiquitin molecule is attached to one lysine residue that is present in the preceding ubiquitin. Several distinct chain-linkage types are possible. The proteasome preferentially recognizes chains where ubiquitins are linked via lysine residue 48, but most other linkage chains can also mediate proteasomal turnover. The process of ubiquitination can be reversed by deubiquitinating enzymes (DUBs). The human genome encodes approximately 80 DUBs that counteract E3 ligase activity [39].

## 2. Ubiquitin–proteasome system components localized to mitochondria

Initial studies of ubiquitin-conjugated proteomes identified several mitochondrial proteins in yeast and mammals as ubiquitination substrates and constituting up to 38% of all cellular ubiquitin conjugates [40–42]. It became apparent that mitochondrial proteins are a substantial part of proteins that are tagged with ubiquitin. Importantly, proteasome impairment results in defects in various mitochondrial functions. A screen for abnormalities in mitochondrial morphology that are caused by the depletion of essential gene products identified proteasome subunits among the hits [43]. These findings revealed associations between mitochondria and the UPS. The mitochondrial localization of various UPS components is another indication of the linkage of these two systems. Several components of the ubiquitination machinery, as well as DUBs, were identified among OM proteins that have both regulatory and quality control functions, including ubiquitin ligases (MARCH5/MITOL and MULAN/MAPL in humans), F-box proteins (FBXL4 in humans and Mdm30 in yeast) and DUBs (USP30 in humans and Ubp16 in yeast) [44–55]. Ubiquitination ligases from other cellular compartments were shown to be recruited to the organelle in response to stimuli/stress, the most notable example of which is PARKIN [56], but this group also includes IBRDC2, FBXW7, FBXO7 and RNF185 in humans, and Rsp5 and Dma1 in yeast [57–62]. Recruitment of the proteasome to the surface of stressed mitochondria was also observed [63–66]. Curiously, Pre6 protein, a non-canonical component of the 20S core particle that can replace Pre9 protein under conditions of stress, was found to localize to the surface of mitochondria, raising the possibility of spatial regulation of proteasome assembly [67,68]. The local presence of both ubiquitin-handling enzymes and proteasomes ensures efficient action with minimal delay.

### 2.1. Ubiquitin–proteasome system regulation of mitochondrial dynamics

**Figure 2.**
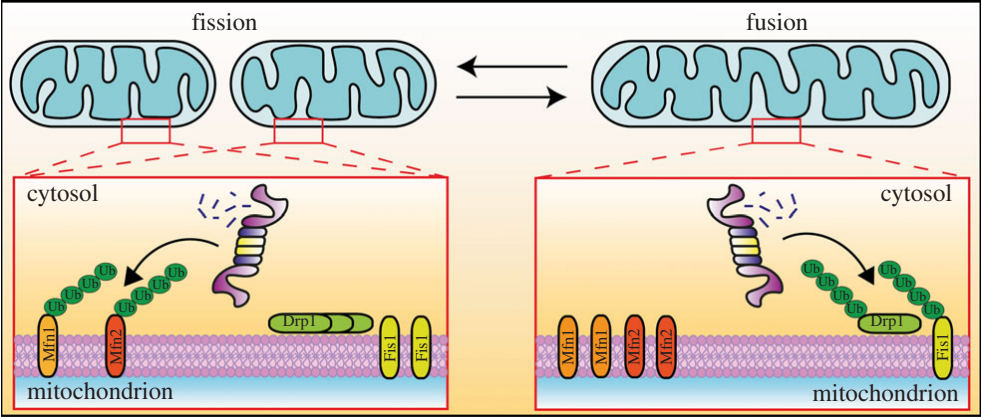
Ubiquitin–proteasome system involvement in the regulation of mitochondrial dynamics. The mitochondrial network in living cells undergoes constant changes that involve organelle fusion and fission (division). Because of the importance of mitochondria, the proper regulation of these processes is critical for cell health. Fusion and fission antagonist processes are regulated by several proteins that promote one or another series of actions. In mammalian cells, during the fission process, Drp1 and Fis1 proteins accumulate on the mitochondrial outer membrane, whereas Mfn1 and Mfn2 proteins are ubiquitinated and degraded by the proteasome. In the process of mitochondrial fusion, the opposite direction is observed, in which Mfn1 and Mfn2 protein levels increase, and Drp1 and Fis1 proteins are directed for proteasomal degradation.

Mitochondria form a highly dynamic network in the cell that is shaped by opposing fusion and fission events (figure 2) [5,12,14]. The rate of these events is balanced by regulatory mechanisms. Any alterations of mitochondrial dynamics result in either hyperfused or fragmented mitochondria. Several key effector proteins of mitochondrial fusion (mitofusins; Fzo1 in yeast and Mfn1 and Mfn2 in humans) and fission (Dnm1 in yeast and Fis1, Drp1, Mff, Mdv1 in humans) are located at the OM. With their domains exposed at the cytosolic side of the membrane, these proteins are directly accessible by the UPS. By selectively removing fusion or fission components, the UPS provides highly effective regulation (figure 2).

In yeast, the Mdm30 complex with Skp1-Cullin-F-box (SCF^Mdm30^) ubiquitin ligase was found to ubiquitinate and promote the degradation of Fzo1 protein [44,52,69]. The *Drosophila melanogaster* homologue of Fzo1 also depends on the proteasome [70]. Similarly, in humans, MARCH5/MITOL E3 ligase influences mitochondrial morphology and affects the levels of Fis1, Drp1, Mfn1, Mfn2, Mcl1 and MiD49 proteins [46,47,71–73]. MULAN/MAPL ligase was shown to influence mitochondrial morphology by reduction of Mfn2 levels [55]. Opposing effects can be attributable to single ligases. This may indicate upstream effectors that modulate their substrate specificity. Nevertheless, a profound influence of the UPS on mitochondrial morphology is conserved among eukaryotic organisms. The UPS-mediated degradation of mitofusins in damaged mitochondria is important for shifting the balance towards fragmentation and preventing fusion with the healthy mitochondrial network, thus supporting the effective separation of dysfunctional mitochondria. A link between the UPS and mitochondrial morphology was further supported by the observation that the expression of proteasomal Blm10 protein dynamically increases in parallel with an increase in mitochondrial biogenesis [74]. Blm10 in yeast and PA200 in humans are proteasome activators that bind to the 20S proteasome and mediate ubiquitin-independent protein degradation in a 19S-independent manner [75,76]. The increase in Blm10 levels was associated with Dnm1 degradation, thus limiting mitochondrial fission during organelle biogenesis.

### 2.2. PINK1 and PARKIN tie the ubiquitin–proteasome system and mitophagy for the effective control of mitochondrial quality

Two proteins, mitochondrial kinase PINK1 and cytosolic E3 ubiquitin ligase PARKIN, are frequently mutated in familial Parkinsonism. They cooperatively control the quality of the mitochondrial population through the selective autophagy of damaged mitochondria. PINK1 is a sensor of mitochondrial fitness. To fulfil this task, PINK1 exploits the canonical presequence-driven mitochondrial import pathway and UPS degradation. In healthy mitochondria, PINK1 is imported into the IM, followed by the proteolytic cleavage of its membrane-bound part. This cleavage releases the remaining catalytic part of PINK1 to the cytosol. Cleaved PINK1 exposes destabilizing amino acid residues at its N-terminus and is rapidly degraded by the UPS [77]. When the presequence import pathway fails because of a decrease in membrane potential or the accumulation of misfolded proteins, PINK1 is routed to the OM to recruit and activate PARKIN [78,79]. PINK1 affects PARKIN in two ways. First, it phosphorylates Ser65 in ubiquitin that is conjugated to OM proteins at basal levels. PARKIN's high affinity for phosphorylated ubiquitin drives its localization to mitochondria. Second, PINK1 phosphorylates PARKIN, activating its ubiquitin ligase activity. Activated PARKIN conjugates further ubiquitins to OM proteins, which are then phosphorylated by PINK1. This forms a positive feedback loop that amplifies the initial signal [80–84].

Shortly after the recruitment of PARKIN, a dramatic increase in ubiquitination is apparent in multiple OM proteins [85]. PARKIN forms ubiquitin chains with linkage types that are characteristic of both autophagy and proteasomal degradation [85,86]. Thus, although dysfunctional mitochondria are degraded by mitophagy, some OM proteins follow a faster degradation route that is mediated by the proteasome [66,87]. This includes the removal of mitofusins to prevent damaged mitochondria from fusing with the healthy mitochondrial network that is a prerequisite for mitophagy [87]. Miro proteins that tether mitochondria to microtubule transportation machinery are also removed by the proteasome upon PARKIN recruitment and activation [88].

Another link to the UPS is provided by the PARKIN ubiquitin-like domain, which has high affinity for the Rpn13 subunit of regulatory particle of the 26S proteasome. This affinity attracts the proteasome to mitochondria and facilitates the proteasomal degradation of selected OM proteins and PARKIN itself [89]. The OM-localized DUB Usp30 negatively regulates PARKIN-mediated mitophagy by removing ubiquitin conjugates from OM proteins. Usp30 controls the steady-state levels of OM protein ubiquitination to prevent accidental autophagy [90].

In the case of prolonged mitochondrial depolarization, a recent study showed that PINK1 and PARKIN can mediate caspase-independent cell death. Notably, this cell death pathway requires proteasome activity [91]. This may indicate sensing a rupture of the OM that results from extensive proteasomal digestion [66]. Along this line, the PARKIN-mediated ubiquitination of internal mitochondrial proteins was observed during the prolonged depolarization of mitochondria [85].

## 3. Mitochondrial proteins facing the cytosol: degradation mechanisms

**Figure 3.**
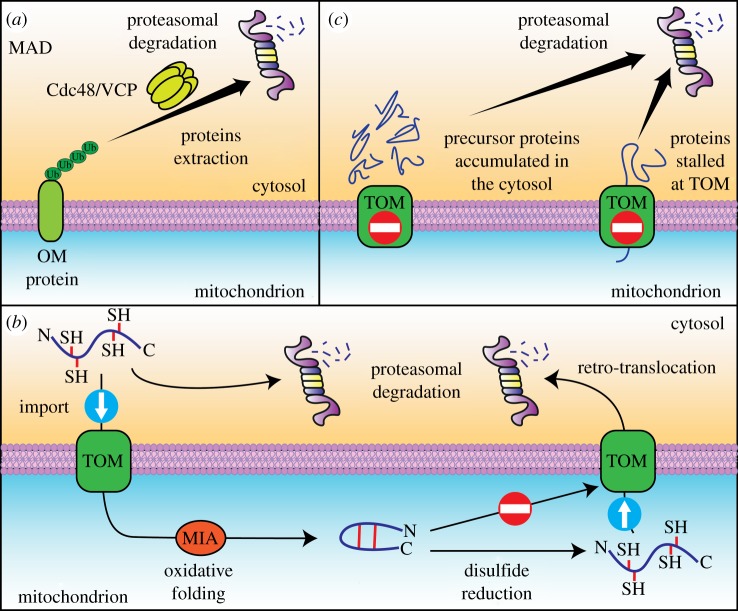
Proteasome-dependent strategies of mitochondrial protein degradation. Mitochondrial proteins are exposed to the proteasomal degradation in several ways. (*a*) The mitochondria-associated protein degradation (MAD) process is homologous to endoplasmic reticulum-associated protein degradation (ERAD). During MAD, mitochondrial outer membrane (OM) proteins are extracted from the organelle through the highly conserved AAA-ATPase Cdc48 (yeast; VCP or p97 in mammals) and directed to proteasomal degradation. (*b*) The proteasomal degradation of mitochondrial proteins also occurs after protein retro-translocation from the mitochondrial intermembrane space. Once proteins are translocated to the mitochondrion through the TOM complex, they are trapped inside the organelle as a result of their oxidative folding that is orchestrated by the mitochondrial import and assembly (MIA) machinery. If the disulfide bonds are not formed or become reduced, then the protein can retro-translocate and be degraded by the proteasome in the cytosol. (*c*) The proteasome is also involved in the degradation of mitochondrial precursor proteins that fail to be transported to the organelle because of defects in protein import or protein stalling on the translocase of the outer membrane (TOM) complex.

Outer mitochondrial membrane proteins are partially exposed to the cytosol, but their proteasomal degradation requires prior extraction from the membrane [92,93]. A molecular machinery that extracts misfolded proteins that are targeted for degradation was first described for the endoplasmic reticulum (ER) [94–96]. Endoplasmic reticulum-associated protein degradation (ERAD) uses an ATPase, Cdc48/VCP/p97, that partners with adaptor proteins (Npl4 and Ufd1) to extract ubiquitinated clients from the ER membrane and allows their proteasomal processing. Recently, Cdc48/VCP/p97 ATPase was found to also extract proteins from the OM in a process called mitochondria-associated degradation (MAD; figure 3*a*) [87,97,98].

In yeast, Vms1 protein was identified as a Cdc48/VCP/p97 partner that can replace Ufd1 in MAD compared with ERAD. Vms1 was found to facilitate the mitochondrial recruitment of Cdc48 during stress [97,99]. Moreover, Vms1 deletion decreased the resistance of yeast to oxidative stress and caused ageing-related mitochondrial dysfunction. Similarly, the knockdown of *daf-16*, a *Caenorhabditis elegans* orthologue of Vms1, reduced the animal's lifespan and oxidative stress resistance. Mitochondria that were isolated from Vms1-deficient cells presented a general increase in ubiquitinated proteins, indicating a possible broad substrate range [97]. However, the function of Vms1 is not essential for the Cdc48-assisted degradation of OM proteins [62,100]. Another component, Doa1, was proposed to also be involved in MAD [62]. Doa1 could be detected as peripherally attached to OM. Strains that lack this protein are sensitized to the increase in mitochondrial reactive oxygen species. Recently, human E3 MARCH5 was found to be involved in both ubiquitination and subsequent steps of OM protein degradation, probably facilitating membrane extraction together with VCP/p97 [101]. Further investigations are needed to better explain the ways in which different adaptors cooperate with Cdc48/VCP/p97 to mediate protein extraction from the OM. Furthermore, an alternative system for OM protein extraction was described. The Msp1/ATAD1 OM protein was identified as an AAA+ ATPase that can mediate the OM extraction of mislocalized ER tail-anchored proteins and thus facilitate their cytosolic degradation [102,103]. This pathway provides a further quality control mechanism that protects against errors in protein targeting and transport.

The repertoire of MAD substrates that have been identified to date is still limited, and currently includes Mfn1, Mfn2 and Mcl1 in humans, and Fzo1, Mdm34, Msp1 and Tom70 in yeast [62,87,97,98]. However, MAD probably provides quality control for OM proteins in general, demonstrated by a recent study on the MAD-mediated degradation of nitrosylated OM proteins [104]. Further studies should provide additional insights into the relationship between different mechanisms of OM protein degradation through the UPS.

## 4. Ubiquitin–proteasome system degradation of internal mitochondrial proteins: protein retro-translocation

All intramitochondrial proteins (i.e. those that are not localized to the OM) are in a proteasome-exclusive location. However, several studies have shown that proteasome inhibition increases protein abundance in mitochondria, supporting either the direct or indirect dependence of such intramitochondrial proteins on the UPS [105–107]. This implies that either mitochondrial proteins at a stage prior to protein import are subject to the UPS, or retro-translocation serves as a mechanism that is a prerequisite of exposing mature proteins to the cytoplasmically located UPS. The retro-translocation process, termed ERAD-L, operates for ER luminal protein degradation [108,109].

The integral IM proteins UCP2 and UCP3 were shown to be degraded in a proteasome-dependent manner following their retro-translocation [64,110,111]. *In vitro* reconstitution experiments indicated that the polyubiquitination of UCP3 that precedes its degradation is mediated by UPS components that co-isolate with mitochondria [111], but remained unclear as to whether this ubiquitination occurs inside the organelle. Interestingly, retro-translocation and degradation required an electrochemical potential across the IM and matrix-localized ATP [64,111], suggesting a mechanism that is energetically similar to mitochondrial protein import.

A recent study revealed that proteins of the mitochondrial IMS, if unfolded, can translocate to the outside of mitochondria [112]. This finding supported the hypothesis that folding into a stable structure traps proteins inside the IMS [19,113,114]. The proper maturation of these proteins in the IMS requires the formation of disulfide bonds that stabilize the protein structure. If protein folding is inhibited, or once a protein becomes unfolded, it can exit the IMS using the same route it used for import, namely the TOM translocase [112,115] (figure 3*b*). IMS protein retro-translocation is size-dependent and more efficient for small proteins. Proteins that escape mitochondria become substrates of UPS degradation. This process provides a quality control mechanism that allows the clearance of misfolded proteins from mitochondria. Importantly, it also provides a means of adjusting mitochondrial protein content in response to cellular needs. During the switch from respiration to fermentation, the levels of IMS proteins that are no longer needed are reduced by this mechanism [112].

Internal mitochondrial proteins frequently undergo proteolytic cleavage of their N-termini. As a result, in some proteins, amino acids residues, which would be destabilizing in the cytosol according to the UPS-mediated N-end rule, become exposed [116,117]. A tempting speculation is that, apart from affecting intramitochondrial protein turnover, such a mechanism would provide rapid proteasomal degradation of these proteins upon their hypothetical release including due mitochondrial rupture. Such a mechanistic framework is used by the PINK1 protein to survey mitochondrial fitness.

## 5. Mitochondrial precursor proteins are cleared by the ubiquitin–proteasome system

From the time of their synthesis until entering the mitochondrial compartment, proteins are under the control of cytosolic quality control mechanisms, including the UPS. Many proteins are ubiquitinated during their translation, including some that are targeted to mitochondria [118,119]. Newly synthesized proteins generally form a substantial fraction of UPS substrates [120]. This probably applies to immature mitochondrial proteins, which remain unfolded before their translocation and maturation inside mitochondria. Indeed, many proteins that are destined to mitochondria, such as apo-cytochrome *c* (Cyc1), ATP5G1, endonuclease G (ENDOG) and classic substrates of the oxidative MIA pathway, are degraded by the proteasome in the case of their import defect or slowdown [106,107,121,122]. Thus, under these conditions, the UPS performs an important surveillance control, preventing accumulation of mitochondrial proteins in an incorrect compartment and decreasing a threat for cellular proteostasis.

Additionally, a plausible assumption is that the import of thousands of protein molecules is not always error-free, and failure probably occurs at basal levels even under physiological conditions. Moreover, protein import is regulated and can be switched off [123,124]. Such circumstances result in unimported mitochondrial proteins in the cytosol and may impact cellular protein homeostasis, thus justifying the need for efficient and selective surveillance and removal mechanisms that are executed by the UPS. Consistent with this possibility, the increase in mitochondrial protein accumulation upon proteasomal impairment that has been observed in previous studies probably results from the inhibition of degradation, followed by the efficient mitochondrial uptake [105–107]. Our study revealed that an entire class of precursor proteins that are destined to the IMS using the MIA pathway are subject to proteasomal degradation [107]. A significant fraction of such precursor proteins is constantly degraded. Importantly, protein removal is not limited to import failure. Inhibition of the proteasome results in an increase in protein import and accumulation in mitochondria, indicating that the proteins rescued from the UPS are functional precursor proteins, and not protein waste. Thus, the mitochondrial import apparatus competes with the UPS for precursor proteins. The kinetics of these competing processes probably favour mitochondria; therefore, successful import dominates under physiological conditions. However, this provides a mechanism of rapid adaptation to the changing needs of mitochondrial biogenesis [107].

Numerous mitochondrial proteins are destined for membrane integration and thus contain TMDs. Hydrophobic properties render TMDs insoluble in the aqueous environment and thus especially prone to misfolding damage. Many mitochondrial proteins are so-called metastable proteins and aggregate if critical concentration thresholds are exceeded [125,126]. The fragile nature of precursor proteins was recently demonstrated by the observation that protein aggregates that accumulate in the cell upon inhibition of the proteasome are enriched for mitochondrial precursor proteins [127]. Moreover, if aggregates are present in the cell, mitochondrial precursor proteins may coaggregate easily limiting their supply for organelle biogenesis [128]. Such proteins require both proteolytic control of their levels and also protective chaperones. The general chaperone proteins Hsp70 and Hsp90 were found to facilitate precursor protein delivery to mitochondria [129]. Factors that shield the TMDs of proteins that are destined to the ER can also bind mitochondrial proteins [122,130,131]. Proteins of the ubiquilin family (UBQLNs in humans) that are normally known as shuttle proteins transferring client proteins for proteasomal degradation were proposed to act as chaperones with high affinity for mitochondrial protein TMDs [122]. UBQLNs were shown to protect mitochondrial precursors from aggregating and to facilitate their mitochondrial import. However, if a precursor protein becomes ubiquitinated, then UBQLNs prevent its mitochondrial import and instead promote proteasomal degradation.

The proteasome degrades proteins individually and protein aggregates are generally accepted to escape proteasomal control. However, a recent study found that UBQLN2 acts with HSP70-HSP110 disaggregase to allow the proteasomal targeting of proteins that are removed from aggregates [132]. This observation and the high affinity of UBQLNs for mitochondrial TMDs raise the intriguing possibility that UBQLNs allow the mitochondrial import of disaggregated precursor proteins. This notion may be linked with another recent observation, in which aggregates that were formed by newly synthesized proteins were often tethered to mitochondria [133].

The ubiquitin ligases that mediate the specific recognition of both soluble and aggregation-prone mitochondrial precursor proteins are unknown. They are probably not mitochondrially localized because this would limit their ability to detect mislocalized proteins in the cytosol. Recently, ubiquitin ligases (Tom1 in yeast and HUWE1 in humans) were shown to guard the level of unassembled ribosomal proteins [134]. Interestingly, a defect of this pathway was reported to increase the formation of protein aggregates that were enriched in both ribosomal and mitochondrial proteins. Whether this demonstrates a role for ubiquitin ligases in mitochondrial precursor degradation or an indirect effect remains to be investigated. Also, the possibility that specific ubiquitination is provided by specialized adaptors that recognize mitochondrial proteins and simultaneously bind E3 ubiquitin ligase should be considered.

The import of most mitochondrial precursor proteins can occur as a post-translational process. However, several studies indicate that protein import in living cells is often a co-translational event that results from the recruitment of messenger RNAs and cytosolic ribosomes to the OM [135–137]. Precursor proteins that are imported into mitochondria co-translationally remain hidden from the UPS. However, in the case of import failure or inefficient ribosomal release, these proteins would require a quality control pathway. Nascent mitochondrial proteins, similarly to other cellular proteins, may stall at ribosomes during their synthesis, especially if translation does not terminate efficiently. This indicates the possible need for the ribosome-associated quality control system (RQC) [138–140]. The RQC system disassembles ribosomes to provide access to stalled nascent peptides that are subsequently targeted for proteasomal degradation by Ltn1 E3 ubiquitin ligase. Such a mechanism was described for ER-stalled nascent peptides at the Sec61 translocon [141]. The N-terminal part of the stalled peptide protruding from the ribosome may initiate the mitochondrial import. In such a case, precursor proteins remain bound to ribosomes and thus cannot complete translocation through TOM translocons. Such translocon clogging is dangerous because it interferes with the import of other precursors. Stalled proteins were shown to be degraded by the proteasome and this required their release from the ribosome [142]. Ribosome quality control can clear clogged translocons and prevent such clogging through the efficient removal of stalled precursor proteins.

Notably, proteins that are imported post-translationally may also block the translocase because of improper, premature folding or aggregation (figure 3*c*). Studies of the mitochondrial proteome showed that precursor forms of intramitochondrial proteins were associated with the OM [15], possibly representing, at least in part, the failure in translocation events. Such proteins may fall under the control of ubiquitination machinery that is present on mitochondria. Quality control mechanisms that are responsible for managing mitochondrial precursor proteins require further investigation.

## 6. Modulation of the ubiquitin–proteasome system by mitochondria

Protein degradation by the UPS is fuelled by ATP, which is used both for ubiquitination and by 19S regulatory particles for substrate insertion into the 20S core particles. Mitochondrial fitness directly influences cellular ATP levels. A drop in ATP levels affects ubiquitination enzymes because ubiquitin-activating E1 enzyme cannot be loaded with ubiquitin [143]. Surprisingly, a decrease in cellular ATP levels can increase proteasome activity [144,145]. Which forms of the proteasome contribute to this increase remains unclear.

**Figure 4.**
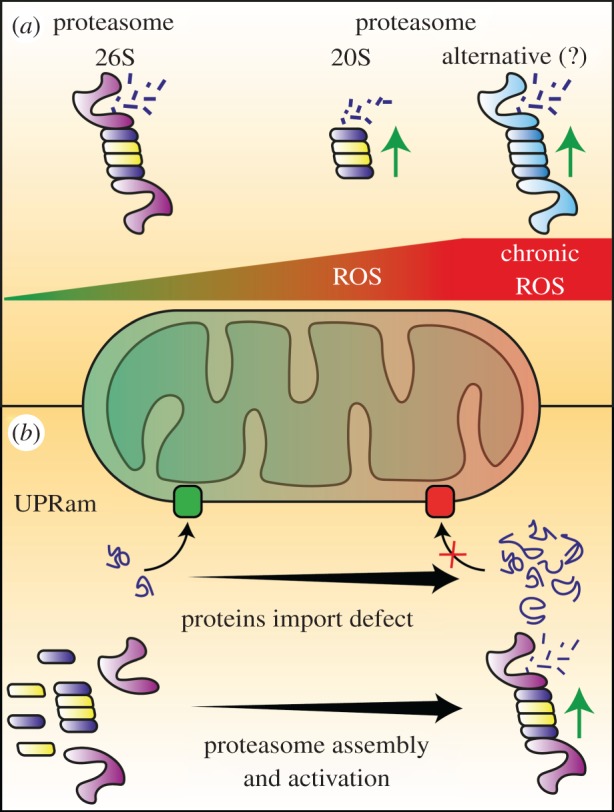
Proteasome activity modulation by reactive oxygen species levels and mitochondrial protein transport impairment. (*a*) An increase in reactive oxygen species (ROS) levels causes higher proteasome activity. A mild increase in ROS levels promotes stronger activity of the 26S proteasome. High ROS levels promote stronger activity of the 20S proteasome. Finally, after a prolonged increase in ROS, more active, alternative proteasome complexes are formed. (*b*) Impairment in mitochondrial protein transport leads to the accumulation of mitochondrial precursor proteins in the cytosol. This increases proteasome assembly and activation in the process of unfolded protein response activated by mistargeted proteins (UPRam).

Mitochondria are the main source of cellular reactive oxygen species. During periods of oxidative stress, damaged proteins accumulate and can threaten cell survival. Mild oxidative stress may increase UPS activity [146,147]. Unexpectedly, both ubiquitination and subsequent degradation by the 26S proteasome were found to be inhibited by reactive oxygen species generated during mitochondrial pathology [148]. Upon bursts of reactive oxygen species, the disassembly of 19S regulatory particles from the 20S core was observed in yeast and human cells [149–152]. Initial 26S proteasome disassembly is accompanied by the transcriptional upregulation of 20S core particle components but also of activators alternative to 19S regulatory particles (e.g. 11S regulatory particle) (figure 4*a*) [153,154]. Interestingly, the 20S proteasome can bind and degrade oxidatively damaged or unfolded proteins [155,156]. Protein degradation by 20S core particles is both ATP- and ubiquitination-independent. This is important because sulfhydryl groups of active sites of E1, E2 and E3 proteins are sensitive to oxidative inactivation [150]. The removal of oxidized proteins by 20S core particles and induction of chaperone expression, including HSP70, allows for 26S proteasome reassembly during cellular adaptations to stress (figure 4*a*). Observed modulation of the proteasome function displays a lot of variation depending on severity and duration of oxidative stress [32,147,157–159].

Until recently, two general cellular mechanisms of the response to mitochondrial dysfunction were described: (i) the retrograde response in yeast and (ii) the mitochondrial unfolded protein response (UPRmt) in higher eukaryotes [160,161]. Both pathways act by increasing the expression of specific genes at the transcription level. The UPRmt provides an increase in internal mitochondrial chaperone and protease levels to intensify protein quality control within the organelle. The retrograde response increases the levels of gene transcripts that maintain the supply of glutamate for biosynthetic pathways during respiratory deficiency. Recently, a post-transcriptional cellular response to mitochondrial malfunction was identified [162,163]. This response is triggered by the cytosolic accumulation of mitochondrial precursor proteins that results from an impairment or slowdown of mitochondrial protein import, called mitochondrial precursor protein over-accumulation stress (mPOS). In one branch of the response, termed the unfolded protein response activated by mistargeted mitochondrial proteins (UPRam), an increase in proteasome assembly is mediated by the assembly chaperone complex, Irc25-Poc4. The increase in assembly is accompanied by an increase in activity of the proteasome, allowing to remove mislocalized proteins (figure 4*b*) [163]. In parallel, a second branch of the response leads to the attenuation and remodelling of cytosolic translation, thus preventing the further build-up of proteotoxic load [162,163]. Interestingly, an increase in proteasomal activity was also proposed to accompany PINK1- and PARKIN-mediated autophagy [91].

Mitochondria strongly influence cellular proteostasis machinery and can globally affect cellular proteome turnover. During mitochondrial defects, different forms of damage can occur simultaneously (e.g. the misfolding and damage of proteins inside mitochondria, cytosolic mislocalization of mitochondrial precursor proteins, a drop in ATP supply, and damage of cellular proteins by reactive oxygen species originating from mitochondria). Thus, the integration of various responses appears to be necessary for the maintenance of cellular homeostasis, revealing an important line of investigation for future research.

## 7. Conclusions and perspectives

The present article provides an overview of the cellular crosstalk between mitochondria and the UPS, with a primary focus on protein biogenesis and turnover. Evidence for a tight connection between mitochondria and the UPS is increasing. Our mechanistic understanding of the interplay between these two cellular systems is still fragmented. Multiple unanswered questions were raised in this review. Moreover, it is still unknown whether precursor protein ubiquitination blocks or generally affects mitochondrial import. The import of ubiquitinated precursor proteins could provide an explanation for ubiquitin-conjugated internal mitochondrial proteins. A recent report described a case of ubiquitination in the mitochondrial matrix, thus opening a new area of study [61]. The internal features of mitochondrial proteins that are required for their specific recognition by the UPS are also unknown. The 20S proteasome, which is capable of degrading oxidatively damaged proteins, might also be involved in the degradation of mitochondrial precursor proteins that remain unfolded before their mitochondrial import. This and other issues often face experimental limitations. Assays that measure proteasome activity do not currently distinguish between different variants of the proteasome that coexist in the cell. Similarly, proteasome inhibitors usually affect the 20S proteolytic core, which is a common element of different proteasome variants. Importantly, the possible inhibition of internal mitochondrial proteases by proteasome inhibitors requires careful consideration.

Ageing causes deterioration of the mitochondrial function. Mitochondrial malfunctions contribute to the ageing process. The UPS is needed to sustain proteostasis, and its activity is modulated by organism ageing [164]. Mild mitochondrial stress or an increase in proteasome activity was shown to be among the triggers of longevity in a *C. elegans* model [165,166]. It is tempting to propose the effects on lifespan are likely to be mediated by a common mechanism that involves the mutual interplay of the two processes, mitochondrial stress responses and proteasomal regulation. As a direct consequence of mitochondrial influence on the cellular proteostasis control, interesting concepts can be raised. In the case of numerous mitochondrial pathologies, cells and organisms will not only experience shortcomings in bioenergetics and metabolic functions assigned to mitochondria, but in addition effects on cellular proteostasis may appear and modulate disease outcomes. Mitochondrial defects will be more devastating on a global scale if not accompanied by the sufficient proteostasis control. *Vice versa*, an imbalance in the cellular proteostasis may result in mitochondrial biogenesis dysregulation, and consequently functional changes, including the increase in reactive oxygen species formation. Research in these areas promises exciting discoveries that will contribute to a better understanding of human health problems associated with ageing societies.
